# Deep-Data-Driven Neural Networks for COVID-19 Vaccine Efficacy

**DOI:** 10.3390/epidemiologia2040039

**Published:** 2021-11-30

**Authors:** Thomas K. Torku, Abdul Q. M. Khaliq, Khaled M. Furati

**Affiliations:** 1 Department of University Studies, Middle Tennessee State University, Murfreesboro, TN 37132, USA; 2 Department of Mathematical Sciences, Middle Tennessee State University, Murfreesboro, TN 37132, USA; abdul.khaliq@mtsu.edu; 3 Department of Mathematics, King Fahd University of Petroleum and Minerals, Dhahran 31261, Saudi Arabia; kmfurati@kfupm.edu.sa

**Keywords:** deep learning, data-driven, COVID-19, ResNet, RNN, vaccination strategy, k-fold cross validation

## Abstract

Vaccination strategies to lessen the impact of the spread of a disease are fundamental to public health authorities and policy makers. The socio-economic benefit of full return to normalcy is the core of such strategies. In this paper, a COVID-19 vaccination model with efficacy rate is developed and analyzed. The epidemiological parameters of the model are learned via a feed-forward neural network. A hybrid approach that combines residual neural network with variants of recurrent neural network is implemented and analyzed for reliable and accurate prediction of daily cases. The error metrics and a k-fold cross validation with random splitting reveal that a particular type of hybrid approach called residual neural network with gated recurrent unit is the best hybrid neural network architecture. The data-driven simulations confirm the fact that the vaccination rate with higher efficacy lowers the infectiousness and basic reproduction number. As a study case, COVID-19 data for the state of Tennessee in USA is used.

## 1. Introduction

The spread of SARS-CoV-2 virus began in Wuhan, China December, 2019 [1]. The virus spread across the globe quickly, affecting many lives and causing deaths, thereby making the World Health Organization (WHO) [1] to declare it a pandemic and naming it COVID-19 [1,2]. Studies on mitigation measures such as social distancing, quarantining, and government shut-downs for controlling the disease show the need for an effective vaccination program. The rate at which the population is vaccinated and its efficacy are two main factors in an effective vaccination program [3]. There is a need for coordinated efforts by all stakeholders such as policy makers, pharmaceutical companies, researchers, epidemiological and health professionals to effectively champion vaccination campaigns for curbing the spread of the disease [4]. Recent studies on vaccine efficacy [5] and vaccine confidence [6] show the importance of an effective vaccination strategy.

Mathematical models have been used to study the dynamics of an epidemic using systems of differential Equations [7,8,9]. The model used in this work is called the SIR model: it divides the population into three groups, namely Susceptible (S), Infected (I), and Recovered (R). The extensions of this basic model have been applied to COVID-19 in recent times [10]. During a pandemic period, the main challenge in this type of model is ascertaining the parameters of the model. Conventional least square method can be used to [11] estimate reasonable constant and time-dependent parameters [12].

Data-driven models that make use of deep learning architectures have been applied to epidemiological models. Raissi et al. [13] used physics informed neural network (PINN) to learn constant parameters of the Influenza model. Kharazmi et al. [12] applied PINN to learn time-dependent parameters of both integer and fractional order epidemiological models. Long et al. [2] showed how PINNis used to learn the time-dependent transmission rate for Susceptible (S), Infected (I), and Recovered (R), and Death (D) (SIRD model), while Grimm et al. [14] presented the effectiveness of using PINN to learn the time-dependent transmission rate of SIR and Susceptible (S), Exposed (E), Infected (I), and Recovered (R) (SEIR) models.

Conventional forecasting techniques such as Auto-Regressive Integrated Moving Average (ARIMA) have been used to make short-term forecasts for confirmed cases of COVID-19 in different countries [15]. Other studies utilized machine learning algorithms, such as Support Vector Machine (SVM), Linear regression, and Exponential Smoothing (ES), to make future predictions of COVID-19 [16]. Recent applications of recurrent neural networks (RNN) with its variant Long Short Term Memory (LSTM) to forecast COVID-19 in Canada, Italy, and USA showed reasonable performance in prediction [17]. The authors in [4] applied LSTM, Bidirectional LSTM (BiLSTM), and Gated Recurrent Unit (GRU) for data-driven simulations of COVID-19 data to different countries. Uncertainties arise from the model, the parameters, and other external factors that impact the accuracy in prediction [11]. There is a need to quantify the uncertainty associated with models [18] for forecasting the trend analysis of COVID-19.

Residual Neural Network (ResNet) is considered, which is capable of learning dynamical systems [19]. The residual connections help to improve accuracy in training and prediction, and are considered as time-stepping techniques modelled after adaptive numerical methods using the concept of integration. Chen et al. [20], in their recent work, showed that the generalized ResNet has the capability of learning intricate unknown dynamic structures of chaotic systems and predicting results with higher accuracy than the standard ResNet structure.

In this paper, a hybrid approach based on residual neural network combined with variants of recurrent neural network for reliable and accurate short-term predictions is considered. The hybrid approach consists of ResNet-LSTM, ResNet-BiLSTM, and ResNet-GRU. The impact of COVID-19 vaccination model with efficacy on each group of population is analyzed. The feed-forward neural network solves the inverse problem which incorporates the epidemiological parameters in the loss function. Error metrics for data-driven simulations are discussed to support the claim of effectiveness for hybrid approach. Furthermore, error metrics and a k-fold cross validation with random splitting are discussed. The paper is organized as follows. Section 2 gives an overview of materials and methods in terms of mathematical model including the Susceptible, Infected, Recovered Vaccine model, non-negativity and boundedness of the model, data preprocessing, error metrics and k-fold cross validation. Section 3 presents the results and discussion of data-driven simulations along with error metrics. Section 4 summarizes the work and presents conclusions.

## 2. Materials and Methods

### 2.1. Mathematical Model

Given the following population groups

*S(t)*: The individuals that are susceptible per time
*I(t)*: The individuals that are infected per time
*R(t)*: The individuals that are recovered per time
*N(t)*: The total population per time
  (1) $$\begin{array}{c} \begin{array}{cc} \frac{dS(t)}{dt} & =-\beta \frac{S(t)I(t)}{N(t)}-v\eta S\left(t\right)\\ \frac{dI(t)}{dt} & =\beta \frac{S(t)I(t)}{N(t)}-\gamma I\left(t\right)\\ \frac{dR(t)}{dt} & =\gamma I(t)+v\eta S(t), \end{array} \end{array}$$
  with S(0)≥0,I(0)≥0,R(0)≥0, N(t)=S(t)+I(t)+R(t), β transmission rate, γ recovery rate, efficacy rate η and vaccination rate *v*. The population per time is assumed to be constant throughout the vaccination regime.

#### 2.1.1. Non-Negativity of the Model

**Theorem** **1.** *If $S_{0}\geq 0,I_{0}\geq 0,R_{0}\geq 0$ then the solutions of the model 1 remain non-negative for all t>0 [21].*

**Proof.**  From model (1)

(2) $$\begin{array}{c} \frac{dS}{dt}\geq -v\eta S. \end{array}$$

Integrating Equation (2) leads

$$\begin{array}{c} S\left(t\right)\geq S_{0}e^{-v\eta t}\geq 0. \end{array}$$

Thus, S(t) remains non-negative for all t>0. Similarly, I(t)≥0,R(t)≥0 all remains non-negative.   □

#### 2.1.2. Boundedness of the Model

**Theorem** **2.** *All the solutions of the model with non-negative initial conditions are bounded and $N\left(t\right)\leq \frac{1}{v\eta }$ for all t>0 [21].*

**Proof.**  The population growth can be written as

(3) $$\begin{array}{c} \frac{dN}{dt}=\frac{dS}{dt}+\frac{dI}{dt}+\frac{dR}{dt} \end{array}$$

It can be seen from Equation (3) that

(4) $$\begin{array}{c} \frac{dN}{dt}=-v\eta N \end{array}$$

Integrating both sides of (4) gives the solution for N(t)

(5) $$\begin{array}{c} N\left(t\right)=\left(N_{0}-\frac{1}{v\eta }\right)e^{-v\eta t} \end{array}$$

Thus, when t→∞, it can be seen

(6) $$\begin{array}{c} N\left(t\right)\leq \frac{1}{v\eta } \end{array}$$

According Theorem 1 and Equation (6),

$$\begin{array}{c} 0\leq N\left(t\right)\leq \frac{1}{v\eta } \end{array}$$

   □

#### 2.1.3. The Basic Reproduction Number $R_{0}$

This refers to the number of people expected to be directly infected by one person who gets the virus [11]. It is defined as

(7) $$\begin{array}{c} R_{0}=\frac{\beta }{\gamma }. \end{array}$$

When the value of $R_{0}<1$, then the disease will disappear at some time in the future. The opposite is true for $R_{0}>1$ with severe effects. A $R_{0}=1$ indicates that the spread of the virus is stable and persistent. In a vaccination regime, the current reproduction number $R_{t}$ is defined as the reproduction number per time. It can be computed as

(8) $$\begin{array}{c} R_{t}=\frac{\beta_{t}}{\gamma_{t}}. \end{array}$$

This depends on a time-varying transmission rate $\beta_{t}$ and recovery rate $\gamma_{t}$. The effective reproductive number $R_{eff}$ is defined as

(9) $$\begin{array}{c} R_{eff}=R_{0}\frac{S}{N}. \end{array}$$

where $R_{0}$ is the basic reproduction number at a period of time and *S* is the number of susceptible individuals and *N* is the population size of a geographical area.

### 2.2. Deep Learning Algorithms

#### 2.2.1. Feedforward Neural Network (FNN)

The feed-forward neural network (FNN) is a type of deep neural network architecture whose node connections are simple and straightforward but not cyclic. The network consists of input, hidden, and output layers. The input layer takes in data and some neurons are applied then propagated forward to the hidden layers, where some linear or non-linear activation functions are applied to transform the data. In the first hidden layer, the weighted sum of the input data through the activation function is computed and propagated through the rest of the hidden layers. A bias term is added to each output from each hidden layer. Thus, the output layer processes the net output from the last hidden layer to produce the desired outcome. If the task is a classification task, then the output layer will produce discrete outcomes but if the task is a regression task, then the output layer will produce a continuous-valued outcome [2,22]. The mathematical formula that transforms the data from one layer to the other is defined as follows [23]:

(10) $$\begin{array}{c} z_{j}^{l+1}=\sum_{i}^{n_{l}}w_{ij}^{l}f_{l-1}\left(z_{j}^{l}\right)+b^{l}, \end{array}$$

where $w_{ij}^{l}$ is the weights between the previous node *i*th and current node *j*th, $b^{l}$ is the bias for the *l*th layer, *f* represents the activation function, $n_{l}$ is the number of neurons, $z_{i}^{l},i=1,\ldots ,n_{l}$, shows the output of the *i*th node in (l−1)th layer. In this work, the activation functions used are the the tangent hyperbolic function

(11) $$\begin{array}{c} \tanh=\frac{e^{x}-e^{-x}}{e^{x}+e^{-x}}, \end{array}$$

and softplus function

(12) $$\begin{array}{c} f\left(x\right)=\ln\left(1+e^{x}\right). \end{array}$$

Different loss functions and optimizers such as Adam or gradient descent method [2] are used to build and train the network to get the output. The difference between the network’s output and the actual data is computed as the error.

**Figure 1:**
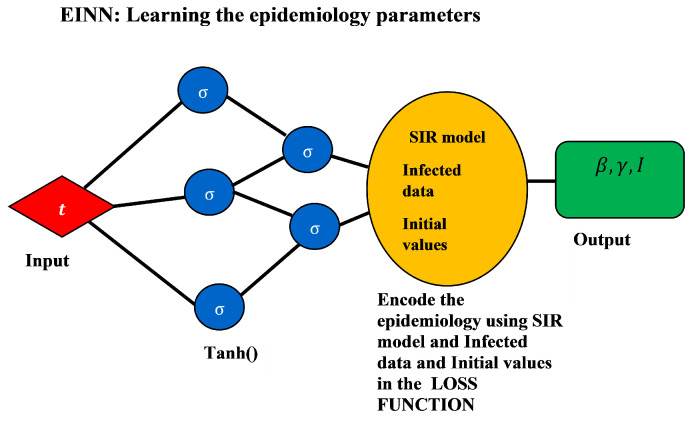
Schematic diagram of Epidemiology Informed Neural Network (EINN). The processed daily infected data at time *t* is passed into the network as seen in red diamond input layer. A hyperbolic tangent activation function is applied to the processed data in the hidden layer. The loss function is defined to incorporate the initial values, data and the residual from the systems of differential equations in (1) in yellow circle. The output layer produces the learned β,γ and the infected group.

**Epidemiology Informed Neural Network (EINN)**
  The epidemiology informed neural network (EINN) is inspired by a physics informed neural network (PINN) [13] which incorporates the epidemiological parameters of the model and initial values into the loss function. . The output from EINN satisfies the differential equations in model (1). This is achieved by encoding the the residuals of the model (1) into the loss function. The method of automatic differentiation [24] is used to compute the derivatives of the output with respect to time for each residual equation. In this study, the mean squared error (MSE) is encoded as the loss function which consists of
  (13) $$\begin{array}{c} Loss=MSE_{data}+MSE_{sir}+MSE_{U_{0}}, \end{array}$$
  where
  (14) $$\begin{array}{c} \begin{array}{cc} MSE_{data}= & \frac{1}{M}\sum_{j=1}^{M}||I\left(t_{j}\right)-\hat{I}\left(t_{j}\right){\left|\right|}_{2}^{2}\\ MSE_{sir}= & \frac{1}{M}\sum_{i=1}^{3}\sum_{j=1}^{M}\left|\right|e_{i}\left(t_{j},\beta ,\gamma \right){\left|\right|}_{2}^{2}\\ MSE_{U_{0}}= & \frac{1}{M}\sum_{j=1}^{M}\left|\right|S_{0}-\hat{S_{0}}{\left|\right|}_{2}^{2}\\ + & \frac{1}{M}\sum_{j=1}^{M}\left|\right|I_{0}-\hat{I_{0}}{\left|\right|}_{2}^{2}\\ + & \frac{1}{M}\sum_{j=1}^{M}\left|\right|R_{0}-\hat{R_{0}}{\left|\right|}_{2}^{2}, \end{array} \end{array}$$
  where the residual $e_{i},i=1,\ldots ,3$
  (15) $$\begin{array}{c} \begin{array}{cc} e_{1}\left(t_{j},\beta ,\gamma \right):= & \frac{dS(t_{j})}{dt_{j}}+\beta \frac{S\left(t_{j}\right)I\left(t_{j}\right)}{N(t_{j})}+v\eta S\left(t_{j}\right)\\ e_{2}\left(t_{j},\beta ,\gamma \right):= & \frac{dI(t_{j})}{dt_{j}}-\beta \frac{S\left(t_{j}\right)I\left(t_{j}\right)}{N(t_{j})}+\gamma I\left(t_{j}\right)\\ e_{3}\left(t_{j},\beta ,\gamma \right):= & \frac{dR(t_{j})}{dt_{j}}-\gamma I\left(t_{j}\right)-v\eta S\left(t_{j}\right). \end{array} \end{array}$$
  The Adam optimizer which is a first order gradient-based optimization is employed to update the networks’ parameters by minimizing the loss function.
  Figure 1 shows the schematic diagram of the epidemiology informed neural network (EINN). The data is preprocessed by using a mini-max scaler factor to allow for smooth training in the neural network. 80 neurons are used for each of the five hidden layers. The Adam optimizer is used in all the data-driven simulations. In order to impose the epidemiological constraints, the Latin hypercube sampling [25] is employed to sample 3000 data points and the spline cubic interpolation is also used to sample 5000 data points. The tangent hyperbolic activation function is applied to all the hidden layers while the softplus activation is applied to the output layer. Implementation of EINN is done in Tensorflow which is run on Python.

The EINN Algorithm 1 for estimating the epidemiology parameters is presented as follows.
**Algorithm 1** **Epidemiology Informed Neural Network (EINN)**1:set input arguments  $t_{j}$, j=1,…,M  vaccination rate *v*  efficacy rate η  epidemiology parameters β, γ  Initial values of the SIR model2:construct and run neural network  specify weights, biases and activation3:compute the residuals  $e_{i}=\hat{y_{i}}-y_{i}$4:calculate loss function  encode the epidemiology
$$\begin{array}{c} Loss=MSE_{data}+MSE_{SIR}+MSE_{U_{0}} \end{array}$$5:train the neural network  set epochs6:return the solution  the epidemiology parameters $\beta^{*},\gamma^{*}$ and infected data $I^{*}$.


In Algorithm 1, a sample code for computing the residuals is given below.

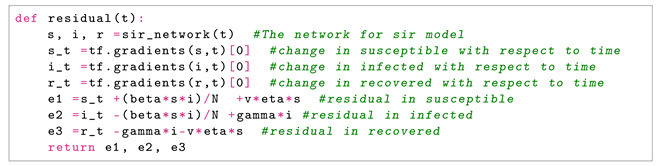



The *tf.gradients* is responsible for the automatic differentiation in Tensorflow which computes the derivatives of the output of the neural network with respect to time. The loss function in Equation (13) is calculated. The network is trained using different epochs. In particular, the epochs is set at 1400 for vaccination rate at 0%, and 800 for the rest of the vaccination rates at 0.5%,1%,2%,3%,6%,10%.

#### 2.2.2. Recurrent Neural Network (RNN)

Recurrent neural networks are networks that are used to learn problems involving sequential data [4]. In this work, all the variants of RNN are implemented in PyTorch.

**Figure 2:**
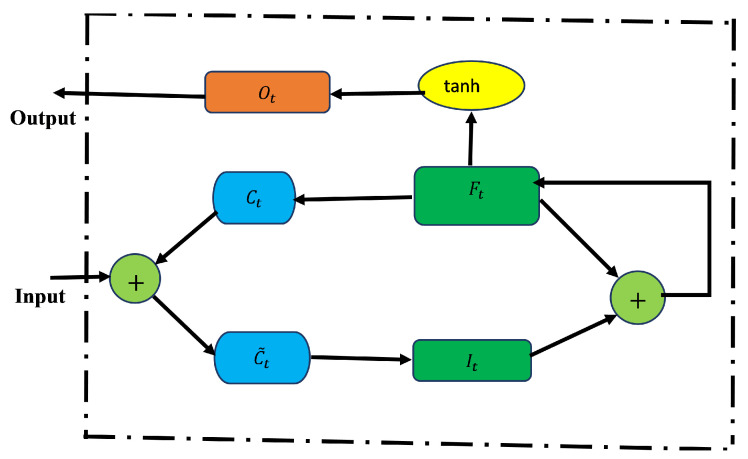
LSTM architecture.

**Figure 3:**
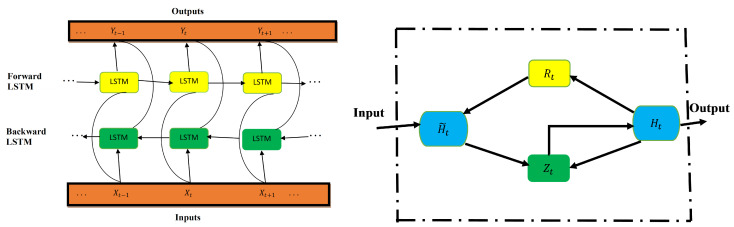
BiLSTM architecture (**left**) and GRU architecture (**right**).

1. **Long Short-Term Memory (LSTM)**
  The long short-term memory (LSTM) is a variant of recurrent neural network (RNN) used to handle sequential data like time series data. It was developed to solve the vanishing gradient problem [26]. The LSTM has three gates that control the flow of information: input, forget, and output gates. These gates have logistic functions that control the weighted sums obtained during training by backpropagation [4]. The cell state manages the input and forget gates. The output comes from either the output gate or hidden state. The unique feature of this network is that it is able to learn long dependencies within the data and able to effectively handle time series data. Given the input data $x_{t}$, and the number of hidden units *h*, the gates of the LSTM can be defined as follows.
  - Input Gate: $I_{t}=\sigma \left(X_{t}W_{xi}+H_{t-1}W_{hi}+b_{i}\right)$
  - Output Gate: $F_{t}=\sigma \left(X_{t}W_{xf}+H_{t-1}W_{hf}+b_{f}\right)$
  - Forget Gate: $O_{t}=\sigma \left(X_{t}W_{xo}+H_{t-1}W_{ho}+b_{o}\right)$
  - Intermediate cell state: ${\tilde{C}}_{t}=\tanh\left(X_{t}W_{xc}+H_{t-1}W_{hc}+b_{c}\right)$
  - Cell State (next memory input): $C_{t}=F_{t}\circ C_{t-1}\circ {\tilde{C}}_{t}$
  - New State: $H_{t}=O_{t}\circ \tanh\left(C_{t}\right)$,
  where
  - $W_{xi},W_{xf},W_{xo},W_{ho},W_{hf}$ are the weight parameters and $b_{i},b_{f},b_{o}$ are the bias parameters of each respective gate.
  - $W_{xc},W_{hc}$ denote the weight parameters, and $b_{c}$ is the bias parameter and ∘ is the element-wise multiplication. The value of ${\tilde{C}}_{t}$ is ascertained from the output of memory cells $C_{t-1}$ and the current time step ${\tilde{C}}_{t}$.
  - ∘ is the element-wise multiplication.
  Figure 2 shows LSTM architecture. The input, forget, and output gates are represented by $I_{t},F_{t},O_{t}$, respectively. The memory cells and memory cell content are denoted, respectively, as *C* and $\tilde{C}$.
2. **Bidirectional LSTM**
  The improved version of the LSTM is bidirectional LSTM (BiLSTM). The network is structured in a way that allows for both backward and forward propagation through the sequential layers [4]. In contrast to LSTM (which only allows for forward pass through the current state), the BiLSTM’s unique advantage is that there is an improvement in accuracy in state reconstruction. This structure combines two hidden states, which allows for information flow from the forward layer to the backward layer. The forward, backward, and output sequences are defined as follows:
  - Forward hidden: ${\vec{H}}_{t}=L\left(X_{t}W_{x\vec{H}}+{\vec{H}}_{t-1}W_{\vec{H}\vec{H}}+b_{\vec{H}}\right)$
  - Backward hidden: ${\overset{\leftarrow }{H}}_{t}=L\left(X_{t}W_{x\overset{\leftarrow }{H}}+{\vec{H}}_{t-1}W_{\overset{\leftarrow }{H}\vec{H}}+b_{\overset{\leftarrow }{H}}\right)$
  - Output: $Y_{t}={\vec{H}}_{t}W_{\vec{H}Y}+{\overset{\leftarrow }{H}}_{t}W_{\overset{\leftarrow }{H}Y}+b_{Y}$.
  where L is the sigmoid function application which is the LSTM unit in the structure of BiLSTM.
3. **Gated Recurrent Unit (GRU)**
  GRU is built to improve the performance of LSTM and reduce the number of its parameters. The input and forget gates from the LSTM model are merged into one gate called the update gate [4]. It is made up of only two gates, update and reset gates, instead of three gates in LSTM. The update gate couples the input and forget gates of the LSTM and the output gate as a reset gate. This gives the GRU an enhanced improvement in LSTM. The relationships among the gates are defined as follows.
  - Update gate: $Z_{t}=\sigma \left(X_{t}W_{xz}+H_{t-1}W_{hz}+b_{z}\right)$
  - Reset gate: $R_{t}=\sigma \left(X_{t}W_{xr}+H_{t-1}W_{hr}+b_{r}\right)$
  - Cell State: ${\tilde{H}}_{t}=\tanh\left(X_{t}W_{xh}+\left(R_{t}\circ H_{t-1}\right)W_{hh}+b_{h}\right)$
  - New state: $H_{t}=Z_{t}\circ H_{t-1}+\left(1-Z_{t}\right)\circ {\tilde{H}}_{t}$,
  where
  - $W_{xr},W_{xz},W_{hr}$ are the weight parameters and $b_{r},b_{z}$ are the bias parameters.
  - $W_{xh},W_{hh}$ are the weight parameters and $b_{h}$ is a bias parameter. The current update gate $Z_{t}$ is a combination of the previous hidden state $H_{t-1}$ and current candidate hidden state ${\tilde{H}}_{t}$.
  Figure 3 presents BiLSTM architecture (left) and GRU architecture (right). In BiLSTM, the forward and backward layers are indicated by yellow and green colors, respectively. In GRU, the reset and update gates are represented by $R_{t}$ and $Z_{t}$, respectively.

#### 2.2.3. Residual Neural Network (ResNet)

This type of deep neural network behaves like first order Euler method [19]. In this work, we cast the ResNet method as an ODE-solver and construct a hybrid approach with traditional recurrent neural networks. Given a deep neural network of the form

$$\begin{array}{c} y^{out}=N\left(y^{in},\theta \right). \end{array}$$

**Figure 4:**
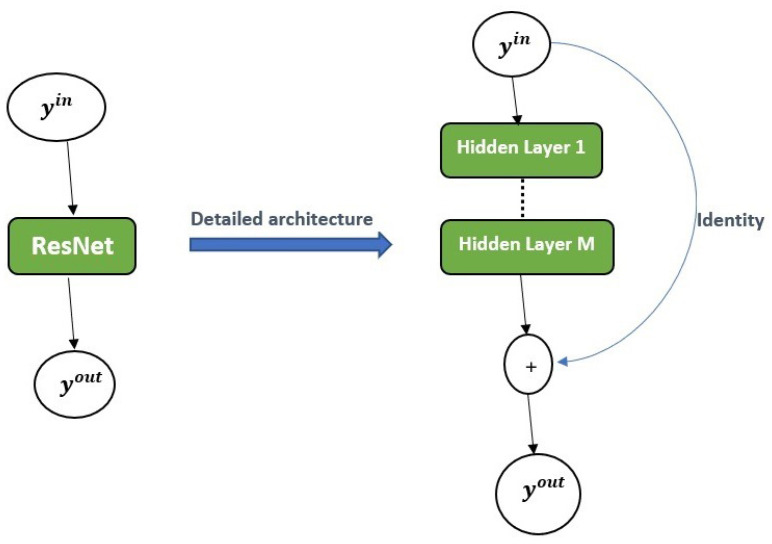
ResNet architecture.

The ResNet is defined as

$$\begin{array}{c} y^{out}=y^{out}+N\left(y^{in}\theta \right), \end{array}$$

where $y^{out}$ is the output, $y^{in}$ is the input (data from solver or fabricated or real measurement), N is the neural network and θ is the parameter of the neural network. The basic structure of the ResNet is given in Figure 4.

### 2.3. Data Preprocessing

The data for this work is obtained from Tennessee Health Department [27]. The available data as of the time of preparing this manuscript is from 12 March 2020 to 16 September 2021. Cumulative cases are used for the training of the EINN where a 7-day rolling mean is applied to the data. In particular, the training data is from 12 March 2020 to 16 December 2020. This is the period of no vaccination program. The data is preprocessed using MinMax scaling. The EINN is implemented in Tensorflow while LSTM, BiLSTM, and GRU as well as k-fold cross validation are implemented in PyTorch.

### 2.4. Error Metrics

In this section, error metrics for data-driven simulations are presented. A survey of the reason and choice of when to use these error metrics is discussed. The metrics used to measure the performance of regression-based models include: Root Mean Squared Error (RMSE), Mean Absolute Percentage Error (MAPE), and Explained Variance (EV) [4]. The values of RMSE are used to compare different models to select the best based on least values [28]. Suppose *y* denotes the real data and $\hat{y}$ is the predicted data from the model.

1. **RMSE:** By the taking the square root of the mean squared error (MSE), the Root Mean Squared Error (RMSE) is obtained and defined as follows:
  (16) $$\begin{array}{c} RMSE=\sqrt{\frac{1}{N}\sum_{i=1}^{N}{\left(y-\hat{y}\right)}^{2}}. \end{array}$$
  This error metric scales the mean squared error (MSE) which serves as a normalizer of large errors. The RMSE serves as a general-purpose error metric and is excellent for numerical predictions [28]. In terms of measuring model accuracy, the RMSE, as a scale-dependent variable, is used to compare errors of prediction from different models or model configurations for a particular variable.
2. **MAPE:** The Mean Absolute Percent Error (MAPE) is the relative error in the mean absolute error.
  (17) $$\begin{array}{c} MAPE=\frac{100}{N}\sum_{i=1}^{N}\left|\frac{y-\hat{y}}{y}\right|\%. \end{array}$$
  The main advantage of using MAPE is that since it is weighted mean absolute error, it is useful for quantile regression. Drawbacks include that it cannot have small values close to zero or zero in the denominator; since percentages cannot exceed 100%, any values high or low cannot be captured; and high negative errors are penalized [29].
3. **EV**-The Explained Variance is the measure of variation in the predicted *y* as explained by the neural network algorithm [4].
  (18) $$\begin{array}{c} EV=1-\frac{Var(y-\hat{y})}{Var(y)}, \end{array}$$
  where Var is the variance between the predicted and the real data. The Explained Variance (EV) is analogous to the co-efficient of determination $R^{2}$ used for linear regression. The EV is ideal for nonlinear regression. When EV gets closer to 1, it means the algorithm predicted the targets correctly.

### 2.5. k-Fold Cross Validation

The k-fold cross-validation is the standard method for evaluating the performance of a deep or machine learning algorithm on a given data [30]. This approach is useful when the available data is small. In practice the typical choice for *k* is either 4 or 5. The procedure consists of a random split of available data into *k* partitions for training *k* identical models. In particular, the training is done on each k−1 partitions and the remaining set (test set) is for evaluating the model. The overall score of the model is determined by the average score from the *k* test scores obtained. Since this paper is about regression model, the error metric used for the scoring each fold is Root Mean Squared Error (RMSE).

Given that a value of k=4 is chosen to perform the k-fold cross validation, the LSTM, BiLSTM and GRU are each run 4 times. This creates 4 identical outcomes for each of the three algorithms. The first three partitions of the data are used for training and the remaining is used for evaluation. The mean squared error (MSE) is used as the loss function. The root mean squared error (RMSE) is computed for each *k* run. These errors are stored in the history of errors.

**Figure 5:**
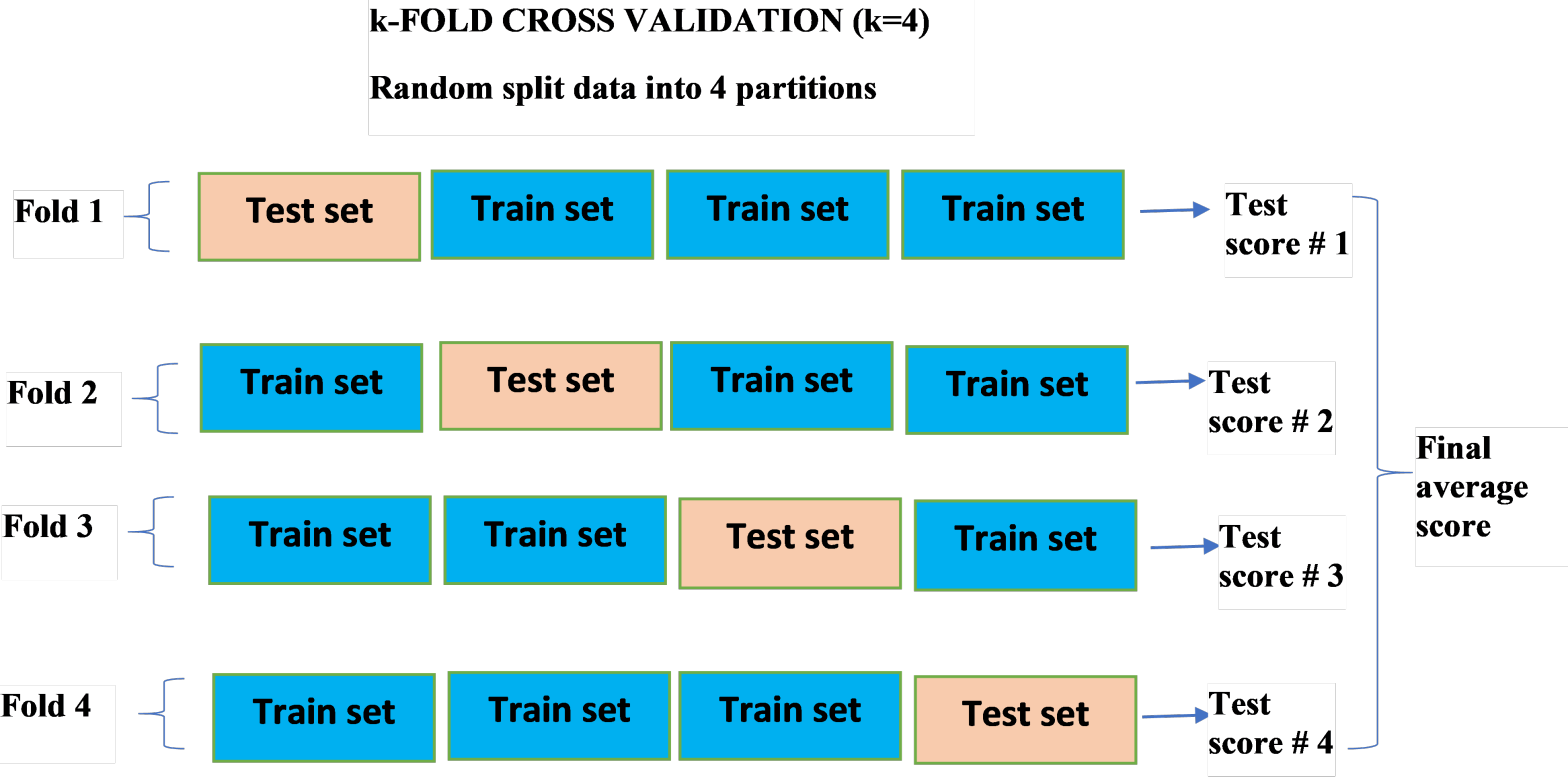
k-fold cross validation. The available data is split into 4 random partitions where each fold has three train sets and one set for evaluating the model. The RMSE is used to score each outcome from training each fold. The final average score is computed from each test score. The standard deviation is also computed.

To ascertain which algorithm performs best, the mean and standard deviations are computed for comparison purposes. In order to have a more reliable comparison, a number of simulations is done using different values of k=5,6,7. The parameter settings for cross validation for real COVID-19 data are: Learning rate 0.01, Epochs 1500, number of hidden layers 1, number of hidden units 16. Figure 5 summarizes above explanation for k-fold cross validation.

### 2.6. Proposed System

**Figure 6:**
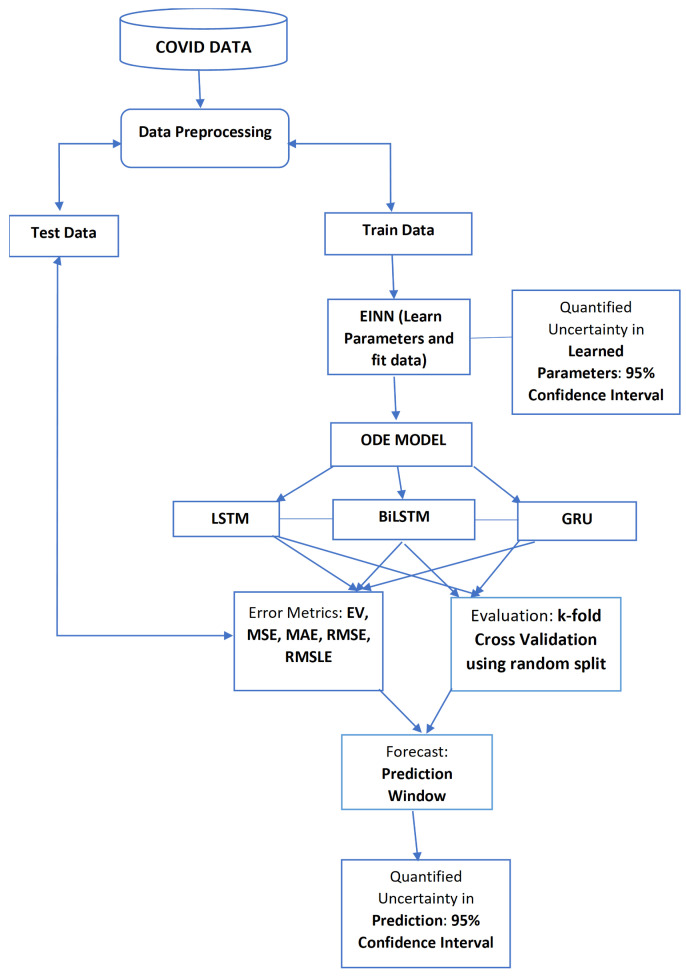
Deep learning forecasting workflow.

Figure 6 gives the overall workflow of the deep learning algorithm for prediction. Available COVID-19 data is obtained from reliable sources. It is randomly split into train and test sets of 80% to 20%, respectively. For EINN algorithm, see Figure 1; the training is done using all days before the start of vaccination. The epidemiology parameters, transmission rate β and recovery rate γ, are learned as well as the infected group. For reliability and accuracy of the learned parameters, the EINN is run 200 times to quantify the uncertainty in the learned parameters by constructing a confidence interval bound using Bootstrap algorithm. The Poisson distribution is used to resample the data [11]. Using different values of vaccination rates, the EINN learns different β and γ. A standard ODE solver is used to produce epidemic outcomes at different vaccination rates using the learned parameters and efficacy rate η. ResNet data is passed into LSTM, BiLSTM, and GRU to have hybrid method ResNet-LSTM, ResNet-BiLSTM, and ResNet-GRU. The error metrics are used to compute the deviations in the test data. A short term prediction into the future is made for 15 days and a confidence interval is constructed. A cross validation is carried out to evaluate the performance of LSTM, BiLSTM, GRU and the hybrid approach.

## 3. Results and Discussion

### 3.1. Data and Parameter Identification

Table 1 gives a statistical summary of Tennessee COVID-19 data, while the skewness is used to check the asymmetry, the kurtosis assesses the flatness of the time series distribution. In general, the value of skewness can either be positive or negative and the kurtosis ranges from −3 to 3. A kurtosis value less than 3 indicates that the distribution is flatter than Gaussian distribution. The dispersion in the distribution can be explained by the other statistics such as quartiles and standard deviation. This summary includes the five number summary, kurtosis, and skewness.

**Figure 7:**
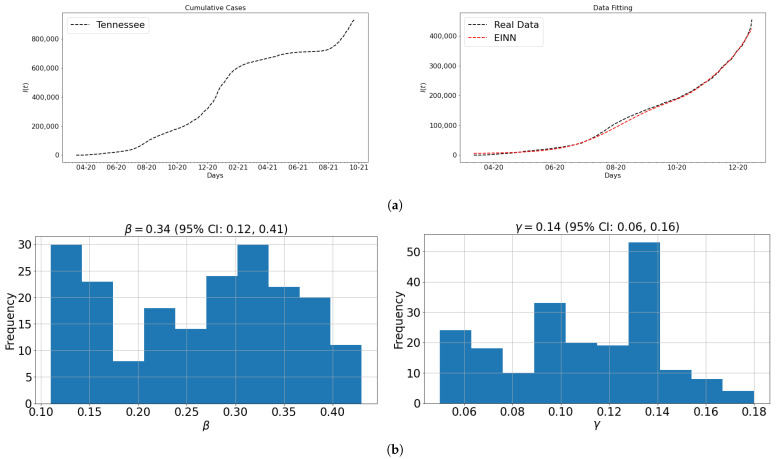
Data Preprocessing and Data Fitting; (**a**) the data (top panel) and data fitting (bottom panel) are graphed; (**b**) the 95% confidence interval is constructed for β and γ for Tennessee.

Figure 7 includes the graphs for real COVID-19 data for Tennessee and the data fitting for learning constant epidemiology parameters using EINN in Algorithm 1. Figure 7 shows the confidence interval constructed for learning the epidemiology parameters using Bootstrap algorithm. In order to have a reliable estimate of the learned parameters, the EINN algorithm is run 200 times. The Poisson distribution is used to replicate similar COVID-19 cumulative data in the Bootstrap algorithm. During each independent run of EINN, the value of each learned epidemiology parameter is stored. The array of each stored parameter is then graphed in the bar graph as distribution.

### 3.2. Data-Driven Simulations

In this section, the EINN algorithm is used to learn different β and γ for different vaccination rates: 0.5%,1%,2%,3%,6%,10%. The underlying assumption is that these rates are assumed the same rate per day for ten months of vaccination. The goal is to analyze the impact of how vaccination with vaccine efficacy makes infections reduce to zero quickly. These values combined with the learned epidemiology parameter values are used to do numerical simulation for model (1) using a standard ODE solver.

**Figure 8:**
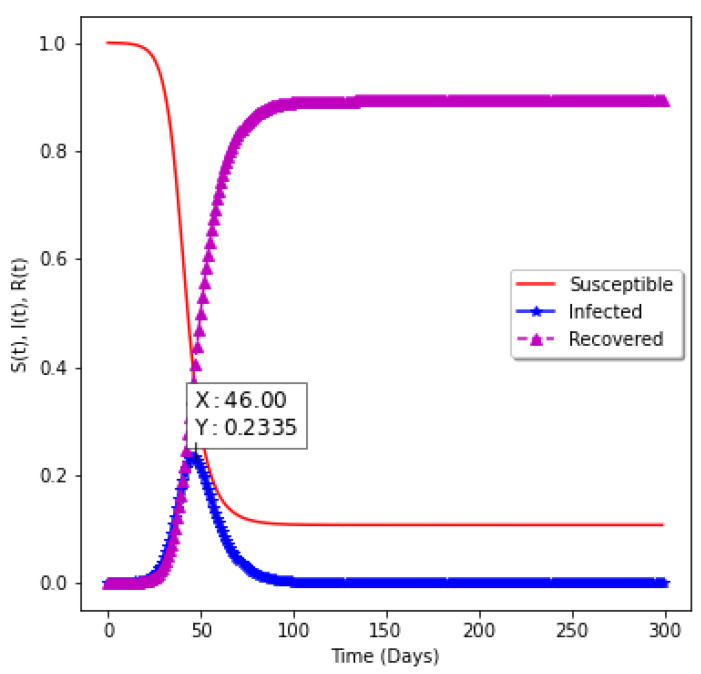
Model without vaccination.

Figure 8 shows the model without vaccination. The epidemiology informed neural network is used to find the epidemiological parameters of the model and the values are passed into the numerical solver to obtain the shape of the graph. It takes 46 days to have a peak assuming there is no vaccination. Approximately 23.35% of the population will be infected with the virus.

Table 2 presents the impact of vaccination with efficacy rate of 80%. In this table, at the different vaccination rates *v*, the EINN learns different $\beta^{\prime }s$ and $\gamma^{\prime }s$ to produce different basic reproduction number $R_{0}$ for the entire period. Equation (7) is used to compute the basic reproduction number. As vaccination rate *v* increases from v=0 to v=0.5%, it leads to the reduction in $R_{0}$ value, from 2.5 to 2.05.

**Figure 9:**
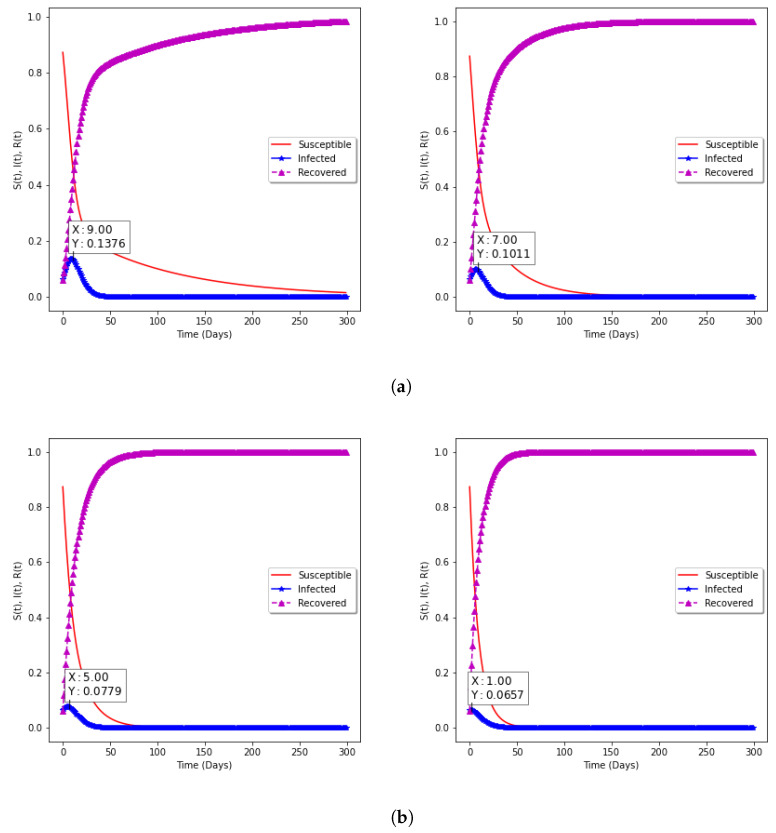
Model with vaccination. (**a**) Vaccination rate of 1% and 3%; (**b**) Vaccination rate of 6% and 10%.

In Figure 9a,b, the model with vaccination is presented. The impact on Susceptible, Infected, and Recovered groups in the population can be seen, while the percentage of the Susceptible individuals decreases as the vaccination rate increases, the percentage of Recovered individuals increases. With the choice of fixed vaccine efficacy η=94%, two points are significant: (**a**) With vaccination rate of 1% and 3%, it can be observed that the percentage of the spread reduces from 13.76% to 10.11% as the rate of vaccination increases from 1.0% to 3%, respectively; (**b**) with vaccination rates of 6% and 10%, the percentage of the spread further reduces to 7.79% and 6.57% for correspondingly.

The impact of vaccination with 94% efficacy is presented in Table 3. It can be observed from the table that for v=0.5%, $R_{0}$ decreased from 2.5 to 2.0. This means that the higher the efficacy rate, the faster the decline in the spread of the virus. This claim is further supported by the fact that when vaccination rate *v* increases to 10%, the $R_{0}$ in Table 2 is 1.38 and the $R_{0}$ in Table 3 is 1.31.

**Figure 10:**
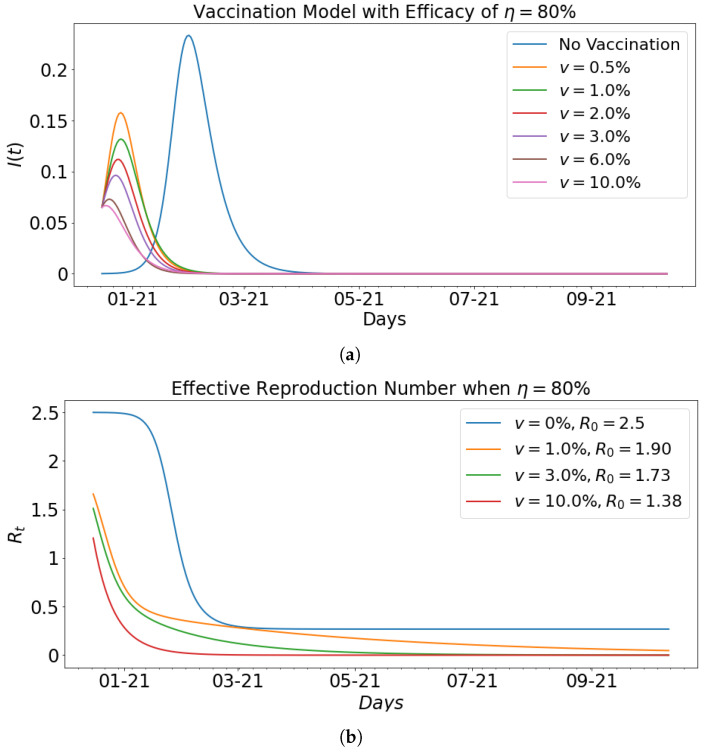
Vaccination model with effective reproduction number when efficacy is η=80%; (**a**), it can be seen that as the vaccination rate *v* increases from 0% to 10%, the level of infectiousness decreases. The effective reproductive number is shown in (**b**) for different vaccination rates *v* with their respective basic reproduction number. As v=0%, $R_{0}=2.5$ and vaccination rate increases from 0% to 3%, the $R_{0}$ decreases from $R_{0}=2.5$ to $R_{0}=1.73$. When the vaccination rate is increased further to 10%, it can be seen that the basic reproduction number reduces drastically to 1.38. This confirms the impact of vaccination in reducing the spread of the virus.

Figure 10 shows the impact of vaccination with fixed efficacy at η=80% on the infected population and the effective reproduction number that corresponds to the basic reproduction number computed in Table 2. In particular, it can be seen that as the respective effective reproduction number for vaccination rates of 0%,1%,3% and 10%. The decrease in the effective reproduction number as shown in the graphs is indicative of the impact vaccination has on the infected group.

**Figure 11:**
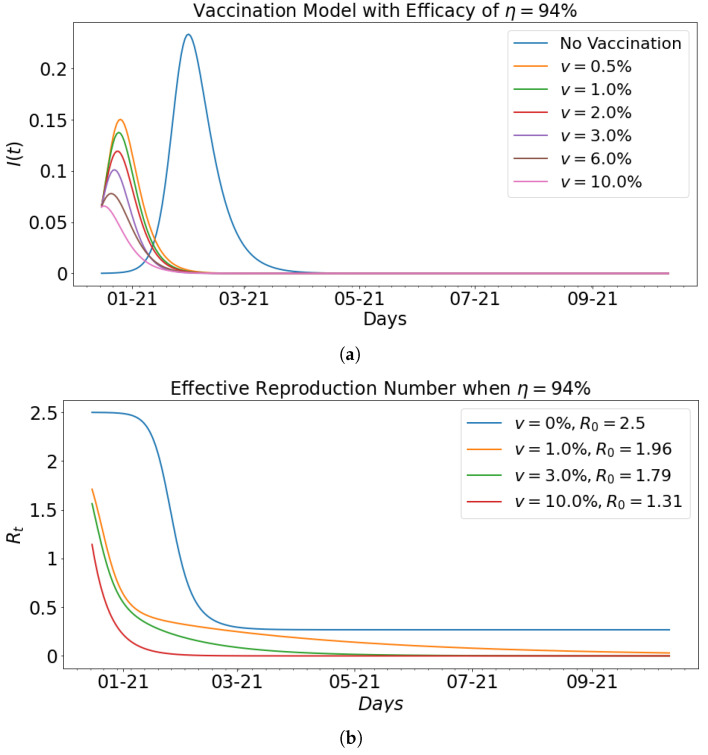
Vaccination model with effective reproduction number when efficacy is η=94%; in (**a**) when the vaccination rate increases from 0% to 10%, the level of infectiousness declines; (**b**) shows the effective reproductive number for different vaccination rates *v* with their corresponding basic reproduction number $R_{0}$. When v=0%, $R_{0}=2.5$, and the vaccination rate increases from 0% to 3%, the $R_{0}$ decreases from $R_{0}=2.5$ to $R_{0}=1.79$. When the vaccination rate is increased further to 10%, it can be seen that the basic reproduction number reduces drastically to 1.31. Vaccination impacts the spread of the virus as seen in this figure.

Figure 11 shows the impact of vaccination with fixed efficacy at η=94% on the infected population and the effective reproduction number that corresponds to the basic reproduction number computed in Table 3. In particular, Figure 11b shows the respective effective reproduction number for vaccination rates of 0%,1%,3% and 10%.

Various data-driven simulations (using the real data and ResNet data) are performed for predicting daily infected cases using LSTM, BiLSTM, and GRU for Tennessee. The LSTM, BiLSTM, and GRU are used to learn these dynamics of infected cases. The parameter settings for data-driven simulations are given in Table 4.

**Figure 12:**
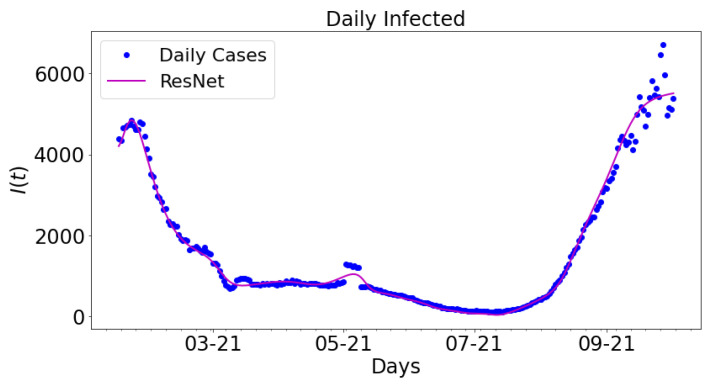
Data-driven simulation for real COVID-19 data and ResNet.

Figure 12 shows the data-driven simulation for learning the infected group using ResNet compared with the daily infected cases. For comparative analysis, the outcome from the ResNet is compared with the daily infected cases. The magenta colored graph is ResNet. The data span a nine-month period: 16 January 2021 to 16 September 2021.

**Figure 13:**
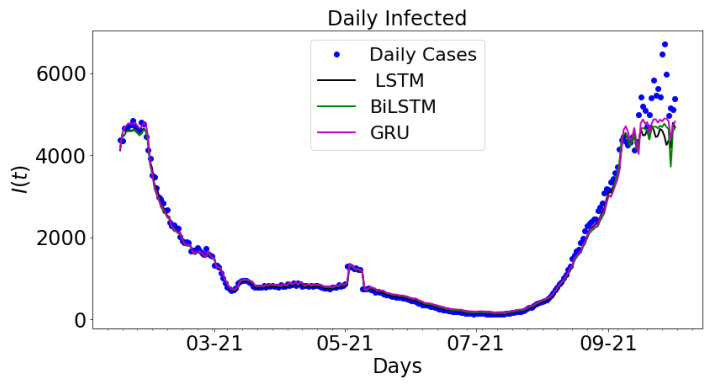
Data-driven simulation for LSTM, BiLSTM, and GRU.

In Figure 13, the output from LSTM, BiLSTM, and GRU are plotted along with the daily infected cases. The real COVID-19 data is used to train the LSTM, BiLSTM, and GRU architectures. The results are plotted along with the daily infected cases. The graph of daily cases is colored blue. The black colored graph shows the output for LSTM, the green colored graph is the graph for BiLSTM, and the magenta colored graph is the graph for GRU. The data spans nine months period: 16 January 2021 to 16 September 2021.

**Figure 14:**
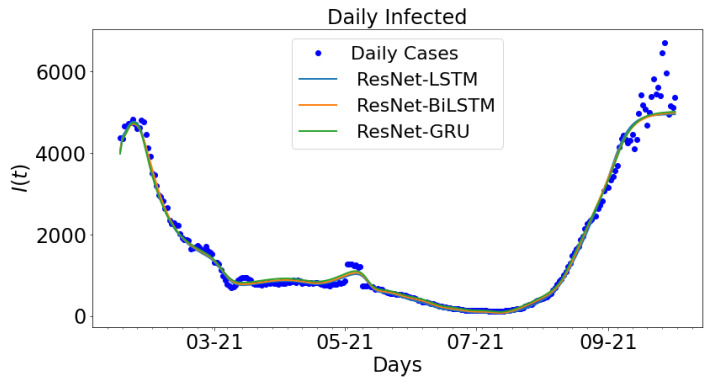
Data-driven simulation for ResNet-LSTM, ResNet-BiLSTM, and ResNet-GRU.

A consideration is made for the hybrid approach: ResNet-LSTM, ResNet-BiLSTM, ResNet-GRU. The outcome ResNet is used as the input data for LSTM, BiLSTM, and GRU. The parameter settings in Table 4 are applied to the different hybrid approaches to generate the ensuring outputs.The outcome for the hybrid approach is shown in Figure 14. The daily new cases are plotted along with ResNet-LSTM, ResNet-BiLSTM, ResNet-GRU. The outcome from the ResNet architecture is called the ResNet data, which is used to train LSTM, BiLSTM, and GRU. The daily cases and ResNet-LSTM are both colored blue while the ResNet-BiLSTM and ResNet-GRU are colored orange and green, respectively. The data spans nine months period: 16 January 2021 to 16 September 2021.

### 3.3. Error Metrics for Data-Driven Simulations

The error metrics from data-driven simulation for real COVID-19 data and for ResNet data from Tennessee are shown below. The data is split into two train and test sets, while the train data is used to train the models, the error metrics are obtained from the difference between the test data of the actual COVID-19 data and the test data from the model.

Table 5 shows the error metrics table for LSTM, BiLSTM, GRU, ResNet-LSTM, ResNet-BiLSTM, and ResNet-GRU. It can be observed that the RMSE values for LSTM, BiLSTM, and GRU are 650.141249, 611.468730, 536.244478, respectively. GRU has the smallest RSME value. Additionally, the MAPE values (relative errors) are 0.159830, 0.150323, 0.131830, respectively, for LSTM, BiLSTM, and GRU. The model with the least MAPE value is GRU. Besides, the corresponding EV values for LTSM, BiLSTM, and GRU are 0.891135, 0.91171, 0.922800. The model with the greatest EV value is GRU. This implies that, using real COVID-19 data, the model with the best error values is GRU. RMSE values for ResNet-LSTM, ResNet-BiLSTM, and ResNet-GRU are 269.863739, 255.473984, 238.104349, respectively. The corresponding MAPE and EV values are 0.065988, 0.062470, 0.058222 and 0.981658, 0.981471, 0.983856. The hybrid approach with the overall best error values in terms of RMSE, MAPE, and EV is ResNet-GRU.

**Figure 15:**
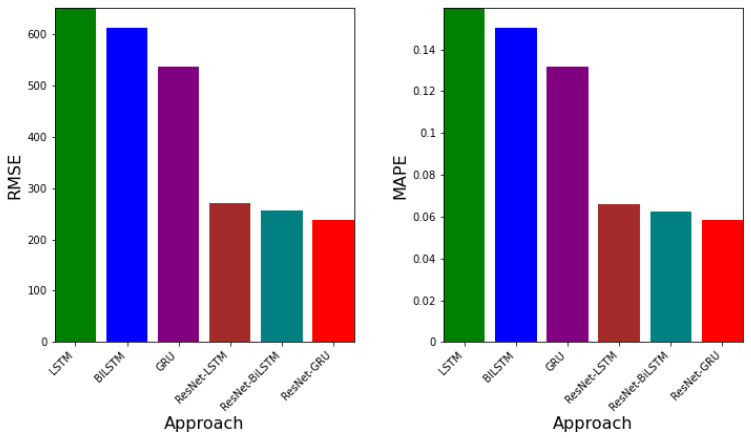
The graph of RMSE and MAPE values for each approach.

Figure 15 presents both the RMSE and MAPE values for each approach. Both error metrics show that the approach with the least error values is ResNet-GRU.

**Figure 16:**
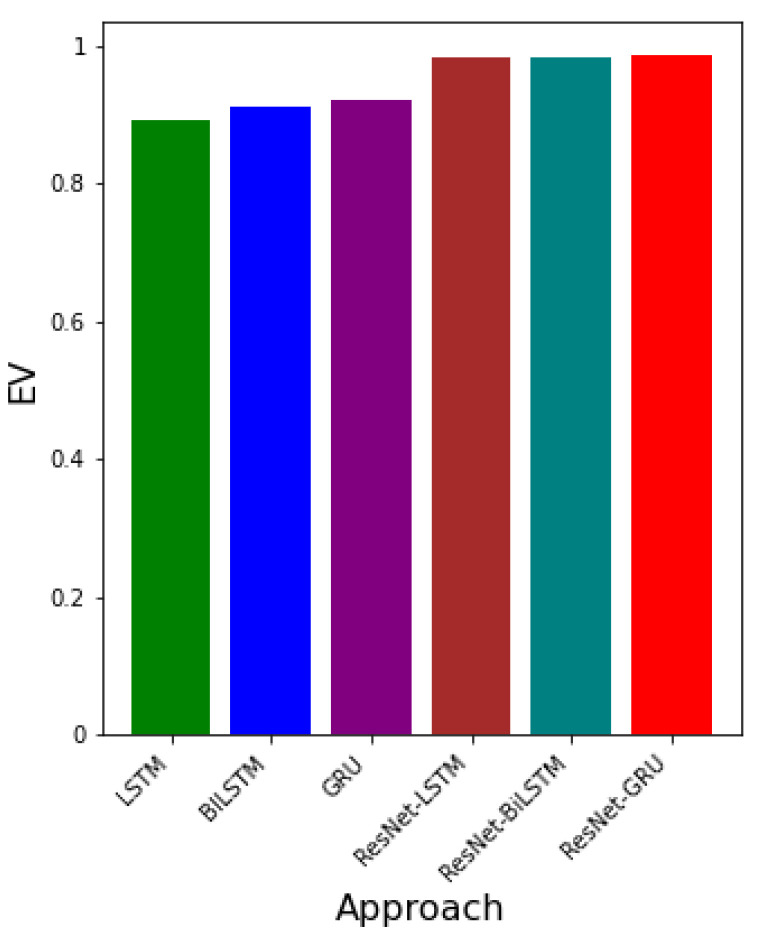
The graph of EV values for each approach.

Figure 16 gives the EV values for each approach. It can be seen that the approach with the greatest EV is ResNet-GRU.

The outcome of the errors obtained from the ResNet-LSTM, ResNet-BiLSTM, and ResNet-GRU, LSTM, BiLSTM, and GRU are presented. The error metrics RMSE, MAPE, and EV are used to determine which algorithm learns the dynamic better. In particular, the RMSE is deployed. Smaller values of RMSE indicate the best algorithm in comparison to the others. The values of EV closer to 1 demonstrates lower variation in the predictive power of the neural network.

### 3.4. k-Fold Cross Validation

**Figure 17:**
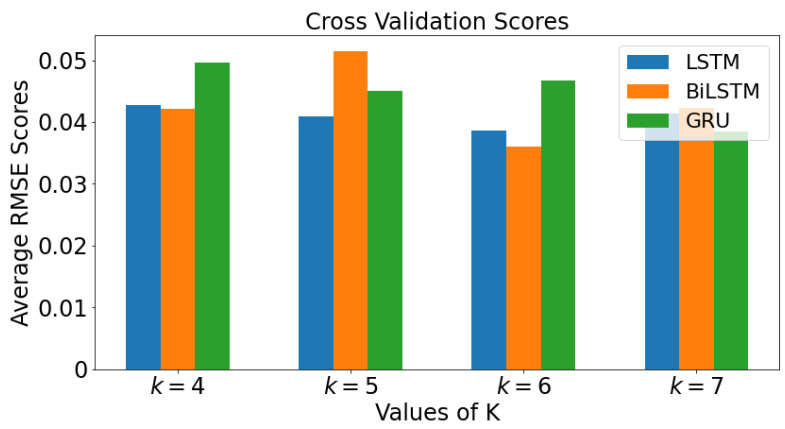
Cross Validation Scores using mean scores for each value of *k* for real COVID-19 data.

The mean values of the RMSE for all four values of *k* are plotted against each value of *k* for each model in a bar graph in Figure 17 for real COVID-19 data. The graph colored blue is LSTM while orange colored and green colored graphs are BiLSTM and GRU, respectively. When k=4, BiLSTM has lowest average RMSE value, followed by LSTM and then GRU. For k=5, LSTM has the lowest average RMSE value, followed by GRU, then BiLSTM. It can be seen that as the value of *k* value increases to 7, the smallest value of RMSE value is GRU, followed by LSTM, then BiLSTM.

**Figure 18:**
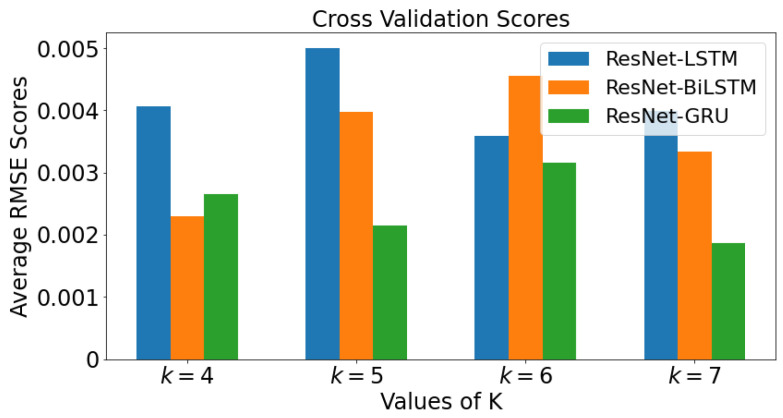
Cross Validation Scores using mean scores for each value of *k* for ResNet data.

Figure 18 demonstrate the mean value of the RMSE. All four values of *k* are plotted against each value of *k* for each model in a bar graph for ResNet data. The bar graph shows the cross validation scores for Tennessee. When k=4, the model with lowest RMSE value is ResNet-BiLSTM, followed by ResNet-GRU, then ResNet-LSTM. With k=5, the model with lowest RMSE value is ResNet-GRU, followed by ResNet-BiLSTM, then ResNet-LSTM. When k=6, it can be observed that ResNet-GRU has the lowest RMSE value, followed by ResNet-LSTM and ResNet-BiLSTM. As the value of *k* increases from 5 to 7, it can be inferred that ResNet-GRU has smallest average RMSE value.

### 3.5. Prediction and Confidence Interval

**Figure 19:**
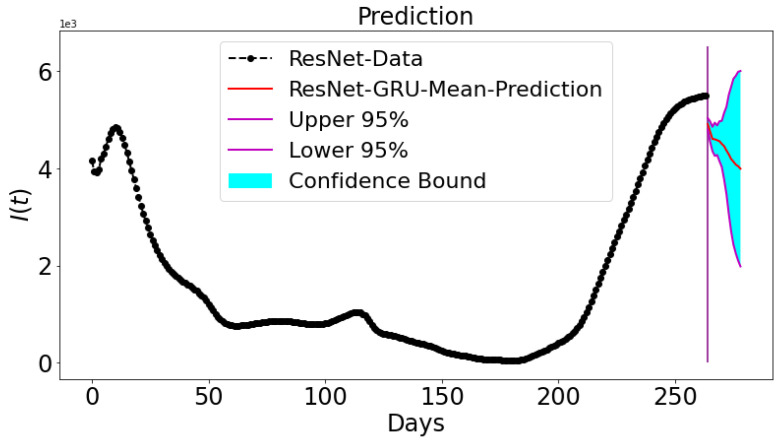
A confidence interval is constructed for a short-term prediction using ResNet-GRU.

Bootstrap algorithm is used to sample a specified number of data samples from a given data and to replicate it for the purpose of constructing a confidence interval. A short term prediction for 15 days into the future (16 September 2021 to 1 October 2021) uses ResNet for Tennessee. The graph of confidence interval for ResNet-GRU predicting 15 days into the future is presented. In Figure 19, the Bootstrap algorithm is used to generate the confidence interval band after 10 independent runs. The lower 95% and upper 95% are given in the graph and the mean prediction is also shown. The Bootstrap algorithm is deployed to run the model 10 times. The Poisson distribution is used to replicate the real COVID-19 data 10 times. For all runs, the mean and standard deviation is computed to ascertain a 95% confidence interval for the future predictions (15 days ahead). The confidence interval bound indicates a reasonable bound for the future predictions.

## 4. Conclusions

In this work, a COVID-19 vaccination model with vaccine efficacy has been developed and analyzed, where the epidemiological parameters of the model were obtained from the inverse problem solved by epidemiological informed neural network. A hybrid approach involving residual neural network with recurrent neural network was implemented. Based on error metric values, the goal of investigating which particular type of the hybrid approach produced reliable future prediction of the infected cases was achieved. In particular, error metric for data-driven simulations demonstrate that ResNet-GRU was the best hybrid approach because of its smallest root mean squared error and mean absolute percentage error values and greatest explained variance value. A k-fold cross validation with random splitting using their average root mean squared error as mean score supported the fact that ResNet-GRU is the best algorithm. Based on quantified uncertainty of 95% confidence interval, the results from short-term prediction demonstrated the overall effectiveness of our approach.

The epidemiological importance of this study was demonstrated in the vaccination model with vaccine efficacy. The model without vaccination revealed a high peak of infectiousness. However, the model with vaccination demonstrated a lower peak of infectiousness. Insights can be drawn from the fact that vaccinating the public using the same vaccination rate per day will curtail the spread of the disease faster. This was confirmed by our study.

## Figures and Tables

**Table 1:** Statistical Summary of COVID-19 Data.

State	Min	Max	STD	Q−0.25	Q−0.5	Q−0.75	Skewness	Kurtosis
Tennessee	1	717,916	279,540.7136	63,503.5	275,565	656,462	0.10058	−1.6981

**Table 2:** Impact of vaccination with (80%) efficacy.

Vaccination Rate (%)	β	γ	$R_{0}$	Infected (%)	Days of Peak
**0.00**	0.35	0.14	2.5	23.35	46
**0.5**	0.45	0.22	2.05	15.78	10
**1**	0.4	0.21	1.90	13.19	10
**2**	0.4	0.22	1.82	11.19	9
**3**	0.38	0.22	1.73	9.63	7
**6**	0.3	0.2	1.5	7.30	4
**10**	0.18	0.13	1.38	6.67	2

**Table 3:** Impact of vaccination with (94%) efficacy rate.

Vaccination Rate (%)	β	γ	$R_{0}$	Infected (%)	Days of Peak
**0.00**	0.35	0.14	2.5	23.35	46
**0.5**	0.44	0.22	2.0	15.04	10
**1**	0.45	0.23	1.96	13.76	9
**2**	0.42	0.22	1.91	11.92	8
**3**	0.43	0.24	1.79	10.11	7
**6**	0.27	0.16	1.89	7.79	5
**10**	0.17	0.13	1.31	6.57	1

**Table 4:** Parameter settings.

Approach	Parameter	Value
LSTM/BiLSTM/GRU	Learning rate	0.01
	Training Epochs	500
	Batch Size	100
	Layers	02
	Features	01
	Hidden units	24
ResNet	Learning rate	0.001
	Training Epochs	200
	Batch Size	45
	Layers	03
	Features	01
	Hidden units	50
Cross Validation	Learning rate	0.01
	Training Epochs	1000
	Batch Size	100
	Layers	01
	Features	01
	Hidden units	12

**Table 5:** Error Metrics.

Approach	RMSE	MAPE	EV
LSTM	650.141249	0.159830	0.891135
BiLSTM	611.468730	0.150323	0.911171
GRU	536.244478	0.131830	0.922800
ResNet-LSTM	269.863739	0.065988	0.981658
ResNet-BiLSTM	255.473984	0.062470	0.983471
ResNet-GRU	238.104340	0.058222	0.983856

## Data Availability

Not applicable.
